# Action potential variability in human pluripotent stem cell-derived cardiomyocytes obtained from healthy donors

**DOI:** 10.3389/fphys.2022.1077069

**Published:** 2022-12-16

**Authors:** A. B. Carvalho, Keyla Cristiny da Silva Coutinho, Raiana Andrade Quintanilha Barbosa, Dilza Balteiro Pereira de Campos, Isabela de Carvalho Leitão, R. S. Pinto, D. Silva Dos Santos, Bruna Farjun, Dayana da Silva De Araújo, Fernanda Cristina Paccola Mesquita, G. Monnerat-Cahli, E. H. Medei, Tais Hanae Kasai-Brunswick, A. C. Campos De Carvalho

**Affiliations:** ^1^ Carlos Chagas Filho Institute of Biophysics, Federal University of Rio de Janeiro, Rio de Janeiro, Brazil; ^2^ National Center for Structural Biology and Bioimaging, Federal University of Rio de Janeiro, Rio de Janeiro, Brazil; ^3^ National Institute of Science and Technology in Regenerative Medicine, Rio de Janeiro, Brazil; ^4^ National Institute of Cardiology, Rio de Janeiro, Brazil

**Keywords:** iPSC (induced pluripotent stem cell), cardiomyocytes, action potential (AP), variability, cell lines, differentiation methods, differentiation batches, healthy donors

## Abstract

Human pluripotent stem cells (PSC) have been used for disease modelling, after differentiation into the desired cell type. Electrophysiologic properties of cardiomyocytes derived from pluripotent stem cells are extensively used to model cardiac arrhythmias, in cardiomyopathies and channelopathies. This requires strict control of the multiple variables that can influence the electrical properties of these cells. In this article, we report the action potential variability of 780 cardiomyocytes derived from pluripotent stem cells obtained from six healthy donors. We analyze the overall distribution of action potential (AP) data, the distribution of action potential data per cell line, per differentiation protocol and batch. This analysis indicates that even using the same cell line and differentiation protocol, the differentiation batch still affects the results. This variability has important implications in modeling arrhythmias and imputing pathogenicity to variants encountered in patients with arrhythmic diseases. We conclude that even when using isogenic cell lines to ascertain pathogenicity to variants associated to arrythmias one should use cardiomyocytes derived from pluripotent stem cells using the same differentiation protocol and batch and pace the cells or use only cells that have very similar spontaneous beat rates. Otherwise, one may find phenotypic variability that is not attributable to pathogenic variants.

## Introduction

Since the advent of human embryonic stem (ES) cells (Thomson et al., 1998) and the reprograming of human adult cells to a pluripotent state by Yamanaka’s group (Takahashi et al., 2007), human pluripotent stem cells (PSC) have been used extensively in multiple areas of biology and medicine (for a review see (Takahashi and Yamanaka, 2016). Methods to differentiate the PSC into cardiomyocytes have been described by different laboratories with efficiencies ranging from 60%–99% (Kattman et al., 2011; Burridge et al., 2011; 2014; Lian et al., 2012), indicating a residual population of non-cardiomyocyte cell types in the most robust differentiation methods, even after metabolic selection (Tohyama et al., 2013). Additionally, there is still variability in the differentiation process depending on donor and donor cell source (Ohno et al., 2013; Sanchez-Freire et al., 2014). Furthermore, although methods to enrich atrial and ventricular cardiomyocyte populations have been described (Lee et al., 2017; Zhao et al., 2019) none ascertain a pure population of chamber specific cardiomyocytes. On top of all these factors the differentiation process leads to an immature phenotype, typical of fetal cardiomyocytes, and although protocols using long term culturing, electric stimulation, mechanical loading, scaffold stiffness, 3-dimensional culturing, epigenetic regulators, metabolic maturation media and neurohormonal stimulation have been developed (Yang et al., 2014; Weinberger et al., 2017; Feyen et al., 2020), combining all these procedures in one single protocol is virtually unattainable.

Considering all these factors it is not surprising that electrophysiologic properties of PSC-derived cardiomyocytes are highly variable. In this article, we report the action potential variability of 780 cardiomyocytes derived from PSC obtained from six healthy donors. One PSC is an ES cell line while the other five lines are induced pluripotent stem (iPS) cells. These six lines were compared as group since they share similar pluripotent properties (Takahashi et al., 2007). We analyze the overall distribution of action potential data, the distribution of AP data per cell line, per differentiation protocol and batch. This analysis indicates that even using the same cell line and differentiation protocol, the differentiation batch still affects the results. This variability has important implications in modeling arrhythmias and imputing pathogenicity to variants encountered in patients with arrhythmic diseases.

## Materials and methods

All data generated or analyzed during this study are included in this published article or in its Supplementary Material.

### Cell lines and culture

The human PSC lines used in this study are described in Supplementary Table S1. HES3 NKX2-5^eGFP/w^ was kindly donated to us by Dr. David Elliot (Elliott et al., 2011). The other cells lines were generated in our laboratory from peripheral blood mononuclear cells using Sendai virus (Thermo Scientific) (Mesquita et al., 2019; Cruvinel et al., 2020) (Kasai-Brunswick et al., 2018). PSC were cultured in mouse embryonic fibroblast feeder layers under standard conditions (Thomson et al., 1998).

### Cardiac differentiation

Two well established cardiac differentiation protocols were used in this study, henceforth referred to as Kattman (Kattman et al., 2011) and Lian (Lian et al., 2012). Both are based on stimulation followed by inhibition of the Wnt pathway using cytokines and/or small molecules. Detailed conditions for each cell line are provided in Supplementary Table S2.

### Action potential recordings

A total of 780 PSC-derived cardiomyocytes were recorded, 138 from ES and 652 from iPS cells (Supplementary Table S3). For Kattman’s protocol, embryoid bodies were digested with collagenase 1 and trypsin-EDTA and plated in Matrigel 2 days in advance to recover from the digestion process. For Lian’s protocol, monolayers were dissociated with trypsin-EDTA and also plated in Matrigel for 2 days. Cells were perfused with Tyrode’s solution (in mM: 140 NaCl, 5.4 KCl, 1.8 CaCl_2_, 1.0 MgCl_2_, 11.0 glucose, 10.0 HEPES, pH 7.4) at 37.0°C ± 1.0°C saturated with oxygen at a perfusion flow rate of 0.5 ml/min. Transmembrane potential was recorded using glass microelectrodes (40–80 MΩ DC resistance) filled with 2.7 M KCl connected to a Microelectrode Amplifier (MultiClamp 700B, Molecular Devices). Amplified signals were digitized (1440 digidata A/D interface, Axon Instruments) and stored in a computer for later analysis using LabChart 7.3 software (ADInstruments). The following parameters were analyzed from at least 10 consecutive action potentials from each cell: maximum diastolic potential (MDP), action potential amplitudes (APA), maximum dV/dt (dV/dtmax), minimum dV/dt (dV/dtmin), action potential duration (APD) at 10 through 90% repolarization, and cycle length. Electrophysiologic data were used from cells having MDP between −100 and −40 mV, APA between 70 and 130 mV, and dV/dt max below 250 V/s.

### Statistics

Statistical analyses were conducted using R (https://www.r-project.org) with RStudio (https://www.rstudio.com) as a visual interface. Raw data, R packages and code used for analyses are provided in the Supplementary Material. Action potential data were compared using Student’s *t*-test, one-way ANOVA followed by Tukey’s post-test to correct for multiple comparisons, or Kruskal-Wallis test. Data were considered statistically significant if *p*-value was below 0.05.

## Results

We first analyzed the overall distribution of resting and action potential properties. Table 1 shows descriptive statistics for 780 PSC-derived cardiomyocytes across six different cell lines, four distinct differentiation protocols and several differentiation batches (18–21 batches/3 cell lines). Supplementary Tables S4–S8 show the same descriptive statistics for all six cell lines individually.

**TABLE 1:** Descriptive statistics for the entire dataset.

Parameter	Min	1st Q	Median	3rd Q	Max	Mean	SD
MDP (mV)	−94	−63.84	−57.53	−51.07	−40.1	−57.89	9.26
APA (mV)	70.08	81.38	90.29	98.59	125	90.63	11.53
dV/dt max (mV/s)	4.4 × 10^3^	1.2 × 10^4^	1.7 × 10^4^	3.1 × 10^4^	2.3 × 10^5^	2.3 × 10^4^	1.8 × 10^4^
dV/dt min (mV/s)	3.7 × 10^2^	9.5 × 10^2^	1.3 × 10^3^	1.7 × 10^3^	4.7 × 10^3^	1.4 × 10^3^	6 × 10^2^
APD10 (ms)	20.29	68.47	83.67	108.55	628.50	97.76	52.45
APD20 (ms)	32.06	101.05	123.4	158.95	671	138.58	61.82
APD30 (ms)	45.82	125.78	151.8	203.93	704.9	176.56	81.26
APD40 (ms)	56.73	141.2	170.7	236.45	752.6	202.32	96.61
APD50 (ms)	64.8	150.9	184.8	254.3	1134	220.1	110.29
APD60 (ms)	73.21	158.97	193.3	266.25	1185	231.74	116.41
APD70 (ms)	82.43	167.10	202	281.07	1205	241.76	119.83
APD80 (ms)	94.61	17.65	212.05	295.32	1224	253.38	123.19
APD90 (ms)	112.8	191.9	241.3	324.9	1290	280.4	136.74
Cycle length (ms)	309.7	1001	1515	1965.5	8634	1597.5	821.39

**FIGURE 1:**
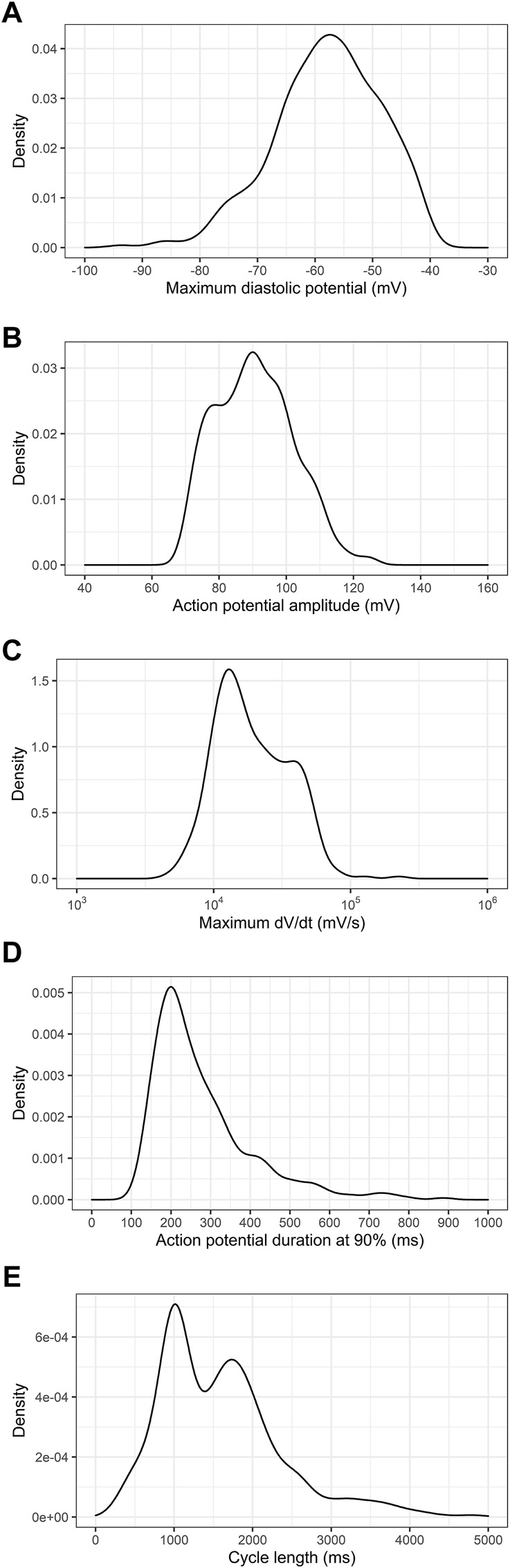
Density distribution of action potential parameters from PSC-derived cardiomyocytes. **(A)** Maximum diastolic potentials (MDP), **(B)** action potential amplitudes (APA), **(C)** maximum dV/dt (dV/dt max), **(D)** action potential duration at 90% repolarization (APD90) and **(E)** cycle length are shown for the whole dataset, comprising 780 electrophysiological recordings.

We next examined the distribution of the electrophysiologic properties of PSC-derived cardiomyocytes obtained from all six cell lines. Figure 1 shows the distribution of maximum diastolic potentials (MDP), action potential amplitudes (APA), maximum dV/dt (dV/dt max), action potential duration at 90% repolarization (APD90) and cycle length across the entire dataset.

**FIGURE 2:**
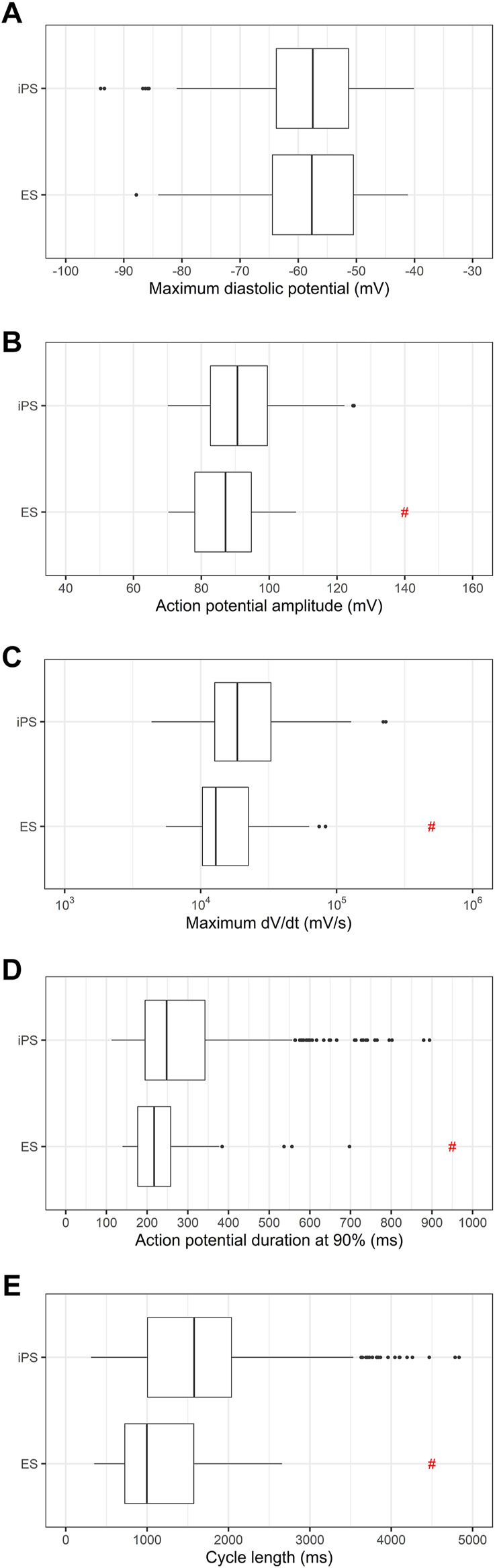
Comparison of electrophysiologic parameters between ES and iPS-derived cardiomyocytes. **(A)** Maximum diastolic potentials (MDP), **(B)** action potential amplitudes (APA), **(C)** maximum dV/dt (dV/dt max), **(D)** action potential duration at 90% repolarization (APD90) and **(E)** cycle length are shown. It is important to note that differentiation protocols varied between the two cell types, and herefore the significance of the differences may be attributable to the varying protocols used for differentiation. # indicates *p* < 0.05 using Student’s *t*-test.

Then, we investigated if the use of ES or iPS could influence in the electrophysiologic parameters of the differentiated PSC-derived cardiomyocytes. Figure 2 shows there is no difference in MDP distribution, but there is significant variability in APA, dV/dt max and cycle length between ES and iPS-derived cardiomyocytes.

**FIGURE 3:**
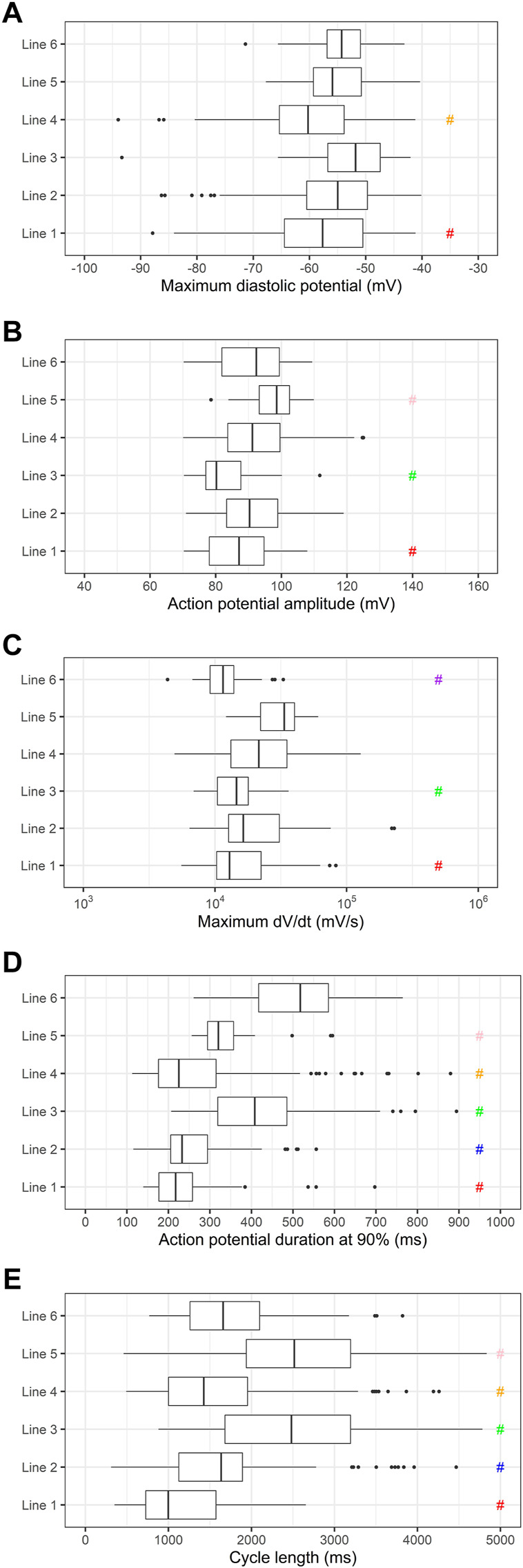
Comparison of electrophysiologic parameters of cardiomyocytes differentiated from individual cell lines. **(A)** Maximum diastolic potentials (MDP), **(B)** action potential amplitudes (APA), **(C)** maximum dV/dt (dV/dt max), **(D)** action potential duration at 90% repolarization (APD90) and **(E)** cycle length are shown. # indicates *p* < 0.05 using one-way ANOVA followed by Tukey’s post test. Significant differences were observed in the following parameters: MDP (red: 1 vs. 3, orange: 4 vs. 2, 3 and 6), APA (red: 1 vs. 2, 4 and 5, green: 3 vs. 2, 4, 5 and 6, pink: 5 vs. 1, 2 and 3), dV/dt max (red: 1 vs. 2, 4 and 5, green: 3 vs. 2, 4 and 5, purple: 6 vs. 2, 4 and 5), APD90 (red: 1 vs. 3, 5 and 6, blue: 2 vs. 3, 5 and 6, green: 3 vs. all lines, orange: 4 vs. 3, 5 and 6, pink: 5 vs. all lines), cycle length (red: 1 vs. all lines, blue: 2 vs. 1, 3 and 5, green: 3 vs. 1, 2, 4 and 6, orange: 4 vs. 1, 3 and 5, pink: 5 vs. 1, 2, 4 and 6).

To investigate the variability in the electrophysiologic properties of the PSC-derived cardiomyocytes from all 6 cell lines individually, we plotted the values of MDP, APA, dV/dt max, APD90 and cycle length, as shown in Figure 3. As expected from the literature, there are significant differences between some of the lines.

**FIGURE 4:**
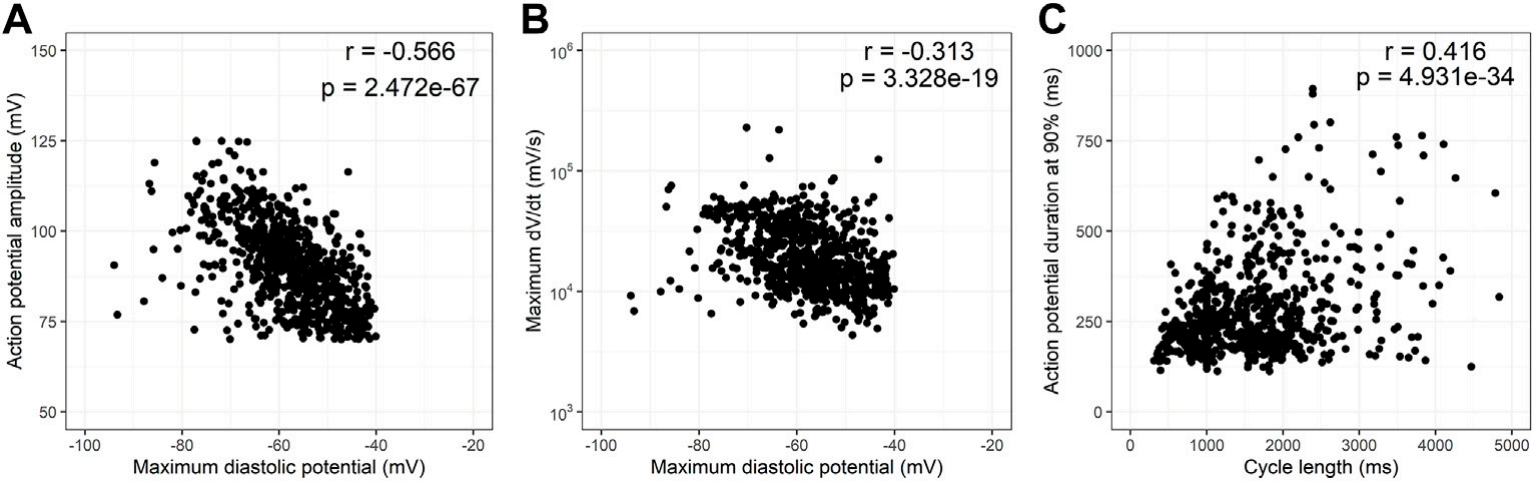
Correlation of action potential variables. Scatter plots of action potential amplitudes (APA) and maximum dV/dt (dV/dt max) as a function of maximum diastolic potential (MDP) are shown in **(A,B)**, respectively. **(C)** shows a scatter plot of action potential duration at 90% repolarization (APD90) as a function of cycle length. All correlations are statistically significant although correlation coefficients are low. Heteroscedasticity increases with cycle length especially above 2,000 ms.

Since MDP is known to influence the action potential amplitude and rate of depolarization, in Figures 4A, B we analyzed the influence of MDP in APA and dV/dt max. Although the correlation coefficients found are low, there is a strong negative correlation between these parameters as expected in cardiac electrophysiology. Figure 4C analyzes the influence of cycle duration in ADP90. There is a strong positive correlation between these parameters but at higher cycle lengths there is clear heteroscedasticity. Supplementary Figure S1 shows the same analysis as Figure 4, but now highlighting the two cell types (ES and iPS, 1a), the four differentiation protocols (1b) and the six cell lines individually (1c). Segmentation by these variables does not differ from the entire dataset.

**FIGURE 5:**
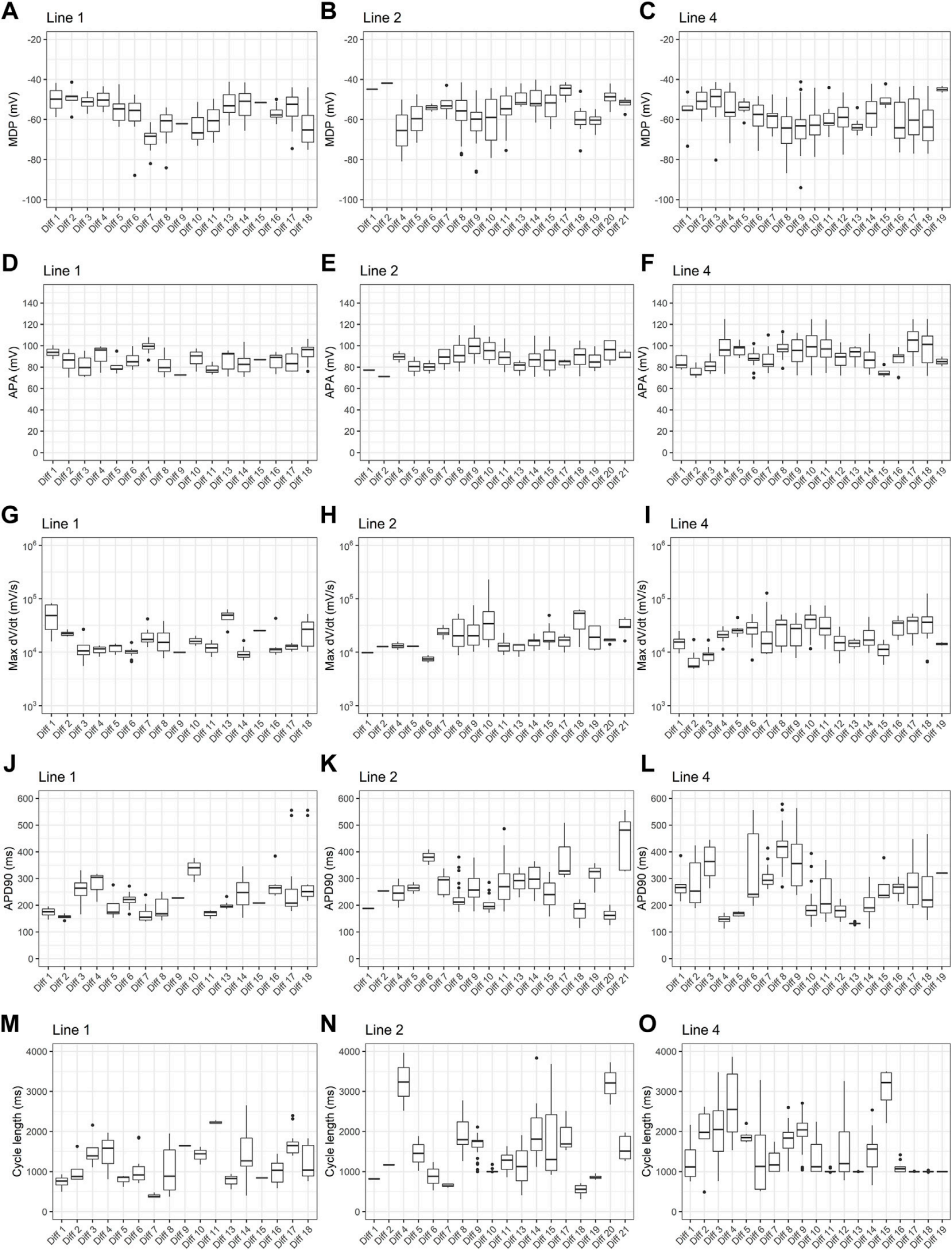
Effect of differentiation batch on action potential parameters. Box plots of **(A–C)** maximum diastolic potentials (MDP), **(D–F)** action potential amplitudes (APA), **(G–I)** maximum dV/dt (dV/dt max), **(J–L)** action potential duration at 90% repolarization (APD90) and **(M–O)** cycle length are shown for three different cell lines. For line 1, all batches used Kattman’s differentiation protocol. For lines 2 and 4, Lian’s protocol was used for all batches.

To investigate the effect of differentiation batch, we analyzed resting and action potential parameters obtained from PSC-derived cardiomyocytes derived from 3 different cell lines submitted to 18–21 differentiation batches. For each cell line, all differentiation batches used the same protocol, but line 1 used Kattman’s protocol and lines 2 and 4 used Lian’s protocol. As shown in Figure 5, across all parameters analyzed, there is considerable variability depending on batch number.

**FIGURE 6:**
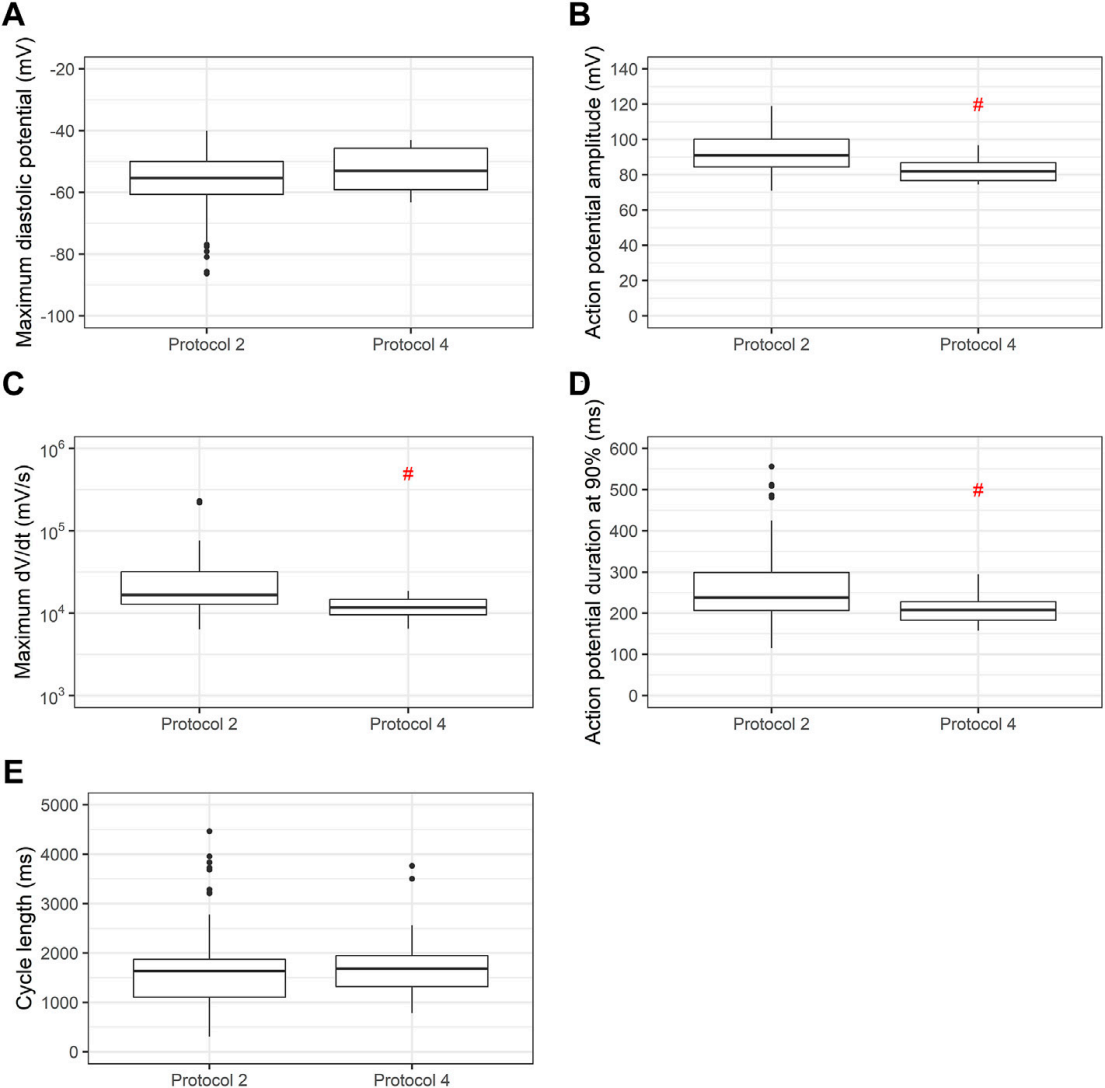
Comparison of action potential variables using two differentiation protocols in the same cell line. Box plots of **(A)** maximum diastolic potentials (MDP), **(B)** action potential amplitudes (APA), **(C)** maximum dV/dt (dV/dt max), **(D)** action potential duration at 90% repolarization (APD90) and **(E)** cycle length for cell line 2 using Lian’s and a commercial protocol. Red # indicates *p* < 0.05 using Student’s t-test.

When we use the same cell line submitted to two distinct differentiation protocols (Lian’s and a commercial protocol) we find significant differences in APA, dV/dt max and APD90, as shown in Figure 6.

**FIGURE 7:**
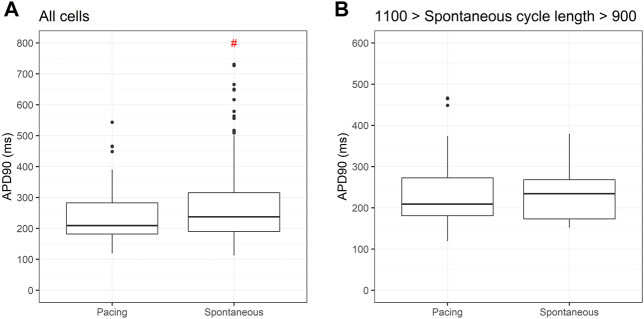
Action potential duration at 90% repolarization (APD90) under spontaneous and pacing conditions. Box plots in **(A)** shows significant differences in APD90 between PSC-derived cardiomyocytes beating spontaneously and paced at 1 Hz. In **(B)**, using only spontaneous cycle lengths varying between 0.9 and 1.1 Hz, similar values for APD90 are obtained under both conditions.

Given the known influence of cycle length in action potential duration we compared APD90 for two cell lines submitted to the same differentiation protocol under spontaneous and paced conditions. Figure 7A shows significantly greater values for APD90 under spontaneous beating when compared to pacing at 1 Hz. If we restrict spontaneous cycle length to 10% variation of the pacing, APD90 is similar between the two conditions.

**FIGURE 8:**
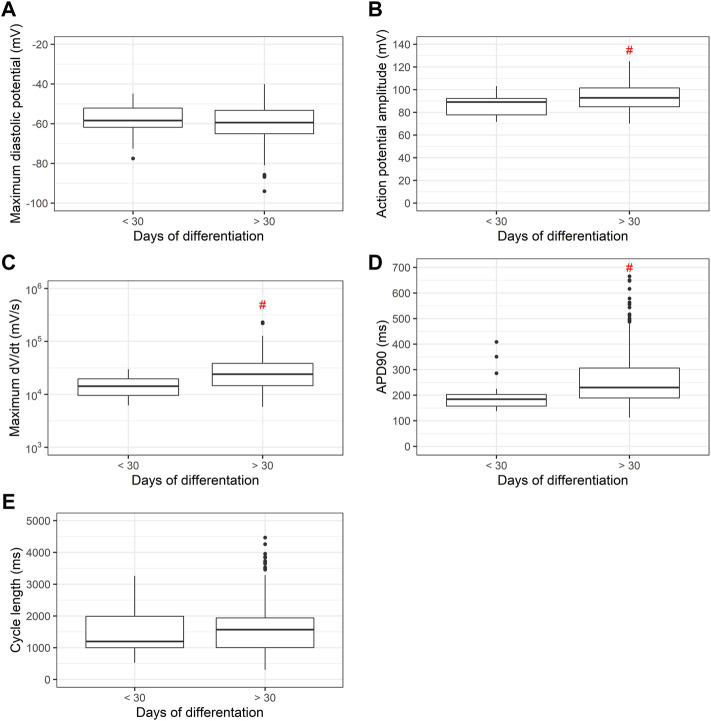
**(A)** Maximum diastolic potentials (MDP), **(B)** action potential amplitudes (APA), **(C)** maximum dV/dt (dV/dt max), **(D)** action potential duration at 90% repolarization (APD90) and **(E)** cycle length as a function of differentiation time. Cardiomyocytes derived from PSC differentiated for less or more than 30 days show significant increases in APA, dV/dt max and APD90 with longer differentiation times.

Since differentiation time is known to produce more mature PSC-derived cardiomyocytes, in Figure 8 we measured MDP, APA, dV/dt max, APD90 and cycle length between PSC-derived cardiomyocytes before and after 30 days of differentiation. After 30 days of differentiation, significant increases in APA, dV/dt max and APD90 are recorded.

## Discussion

The use of PSC in disease modelling has become widespread due to the uncertainties of animal models as faithful models of cardiac human diseases (Saura et al., 2022; Houser et al., 2012). In particular, the use of cell types derived from PSC extracted from diseased patients (disease specific PSC) avoids the costly and risky nature of biopsies. Therefore, PSC have attained prominence for disease modeling, especially in monogenic diseases, but also in more complex diseases as the concept of disease in a dish has advanced not only in modeling but also in drug screening. Non-etheless, the use of PSC for disease modeling has limitations that have been addressed in many reviews (Saha and Jaenisch, 2009; Soldner and Jaenisch, 2012; Sharma et al., 2020; Horwitz et al., 2021).

In cardiac pathophysiology, PSC-derived cardiomyocytes have been intensively used to model inherited arrhythmic cardiac diseases since the seminal works of Moretti et al. (2010), Itzhaki et al. (2011) in which they compared the electrophysiologic properties of cardiomyocytes derived from patients with long QT syndrome (LQTS) to those of healthy controls. Since then, the field has become prolific in publications where 1 or a few disease cell lines are compared to 1 or a few controls cell lines. These comparisons have obvious limitations since the diverse genetic backgrounds may introduce confounding factors that influence the observed phenotype. Using control cells from the families of affected patients may reduce, but not abolish these confounding factors and the field has become stricter about attributing the altered phenotype to the diseased cells. Currently, isogenic cell lines, where the variant encountered in the diseased cell is corrected by gene editing technologies in monogenic diseases should be mandatory to investigate the pathogenicity of variants. The availability of CRISPR based techniques to perform gene editing in PSC has made this possible (Doudna and Charpentier, 2014).

In this article we used control cell lines from six healthy donors and show that there is great variability in physiologic properties of the PSC-derived cardiomyocytes differentiated from these lines, as shown by others (Sanchez-Freire et al., 2014; Sala et al., 2016; Mannhardt et al., 2020). Furthermore, we show that the electrophysiologic data for these six cell lines is also dependent on the differentiation protocol used and on differentiation batch. Similar results have been described by Mannhardt et al. (2017), Mannhardt et al (2020) when measuring contractile properties in control iPS-derived cardiomyocytes and Huo et al. (2016) when evaluating iPS-derived cardiomyocytes from two commercial suppliers. We also show that cycle length influences APD90, and great dispersion is observed with periods above 2,000 ms, indicating that pacing should be preferred when comparing APD from distinct cell lines. Furthermore, electrophysiologic properties of PSC-derived cardiomyocytes are influenced by time after differentiation. Significant increases in APA, dV/dt max and APD90, when comparing differentiation protocols with less or more than 30 days, indicate that a more mature phenotype can be achieved with longer differentiation periods.

Age, sex, cell source and race are other variables that can influence the molecular and physiologic parameters of PSC-derived cardiomyocytes. D’Antonio-Chronowska et al. (D’Antonio-Chronowska et al., 2019) reported the influence of the X chromosome on the differentiation trajectories of human iPS into cardiomyocytes. Pianezzi et al. (2020) suggest that iPS derived from cardiac sources differentiate into more mature cardiomyocytes. But race has not been reported to influence calcium transient kinetics or beat rate in iPS-derived cardiomyocytes by Schaniel et al. (2021) when generating a library of iPS from diverse healthy human individuals. Age does not seem to influence the reprograming of cells to a pluripotent state (Lapasset et al., 2011), and Schaniel et al. (2021) have not reported differences in the function of cardiomyocytes derived from iPS obtained from healthy patients with ages ranging from 22 to 61 years. Our data concerning these variables is limited since we used three cell lines from male and three from female donors, with a large age span, and except for the ES, all reprogrammed from erythroblasts.

An important point to be considered is if the variability here reported is also present in bona-fide cardiomyocytes isolated from adult hearts. Here we are restricted to animal models for comparisons, due to the obvious ethical barriers related to obtaining cardiac tissue from healthy humans. Using ventricular slices of adult mouse hearts, Halbach et al. (2006) report the variability in resting membrane potential (RMP), APA and APD90 at a fixed stimulation frequency, and observe significant differences in APA and APD90 if stimulation frequency varies by 5-fold. Using guinea pigs heart slices, Bussek et al. (2009) also reported the variability in RMP, APA and APD90 in 59 recordings from left ventricular slice preparations. In Supplementary Table S9 we list the standard deviations found in six articles using rat, mouse and guinea pig cardiomyocytes for RMP/MDP, APA, dV/dt max and APD90, although with a limited number of recordings. As shown in the Table, standard deviations are similar between PSC-derived and animal cardiomyocytes for RMP/MDP, APA and dV/dt max. However, standard deviation is considerably higher in APD90 of PSC-derived when compared to animal cardiomyocytes. Since APD90 is a critical parameter for the modeling of arrhythmic events *in vitro*, this variability should be considered when inputting evidence of pathogenicity using this type of functional assay.

We conclude that even when using isogenic cell lines to ascertain pathogenicity to variants associated to arrythmias one should use cardiomyocytes derived from PSC using the same differentiation protocol and batch and pace the cells or use only cells that have very similar spontaneous beat rates. Otherwise, one may find phenotypic variability that is not attributable to pathogenic variants. Al-Owais et al., 2021, Howlett et al., 2022, Saito et al., 2005, Tan et al., 2014.

## Data Availability

The original contributions presented in the study are included in the article/Supplementary Material, further inquiries can be directed to the corresponding author.

## Supplementary material

SUPPLEMENTARY FIGURE S1 Correlation of action potential variables. The upper row shows scatter plots of action potential amplitudes (APA) as a function of maximum diastolic potentials (MDP) for different cell types **(A)**, differentiation protocols **(B)** and cell lines **(C)**. The center row shows scatter plots of maximum dV/dt (dV/dt max) as a function of MDP for different cell types **(D)**, differentiation protocols **(E)** and cell lines **(F)**. The bottom row shows a scatter plot of action potential duration at 90% repolarization (APD90) as a function of cycle length for different cell types **(G)**, differentiation protocols **(H)** and cell lines **(I)**.
