# Supplementary material for: Action potential variability in human pluripotent stem cell-derived cardiomyocytes obtained from healthy donors
Source: Front Physiol. 2022 Dec 16;13:1077069. doi: 10.3389/fphys.2022.1077069 (PMC9800870; doi:10.3389/fphys.2022.1077069)
Supplement: Supplementary file 1 [file DataSheet2.PDF]

```
##################################
   #Action potential analyzes#
##################################


## Load R packages, filter, clean and organize data ##

library(tidyverse)
library(grid)
library(gridExtra)
library(gtable)
library(car)
library(psych)
library(scales)

data <- read.csv2('ActionPotentialData2019Controlsonly.csv')
data$Cell.line <- ordered(data$Cell.line, levels = c('Line 1', 'Line
2', 'Line 3', 'Line 4', 'Line 5', 'Line 6'))
data$Diff.ID <- ordered(data$Diff.ID, levels = c('Diff 1', 'Diff 2',
'Diff 3', 'Diff 4', 'Diff 5', 'Diff 6', 'Diff 7',
                                                     'Diff 8', 'Diff
9', 'Diff 10', 'Diff 11', 'Diff 12', 'Diff 13',
                                                 'Diff 14', 'Diff
15', 'Diff 16', 'Diff 17', 'Diff 18', 'Diff 19',
                                                 'Diff 20', 'Diff
21'))
data$Day.0 <- as.Date(as.character(data$Day.0))
data$Recording.date <- as.Date(as.character(data$Recording.date))
data <- mutate(data, Days.of.diff = difftime(data$Recording.date,
data$Day.0, units = c('days')))
data$Days.of.diff <- as.numeric(data$Days.of.diff)
data <- mutate(data, Group.days = cut(data$Days.of.diff, breaks =
c(-Inf,29,Inf), labels = c("<30",">30")))
data <- mutate(data, Period.ms = Period*1000)
data <- mutate(data, Period.sqrt = sqrt(Period.ms))
data <- mutate(data, logAPD90 = log(APD90))
subset <- subset(data, MDP < -40 & MDP > -100 & APA < 130 & APA > 70
& Max.dV.dt < 250000)


## Descriptive statistics for the entire dataset ##

data.frame(table(subset$Cell.line))
data.frame(table(subset$Cell.type))
data.frame(table(subset$Diff.protocol))
data.frame(table(subset$Days.of.diff))
data.frame(table(subset$Group.days))

summary(subset$MDP)
sd(subset$MDP)
summary(subset$APA)
sd(subset$APA)
summary(subset$Max.dV.dt)
sd(subset$Max.dV.dt)
```

```
summary(subset$MaxNegSlope)
sd(subset$MaxNegSlope)
summary(subset$APD10)
sd(subset$APD10, na.rm=TRUE)
summary(subset$APD20)
sd(subset$APD20, na.rm=TRUE)
summary(subset$APD30)
sd(subset$APD30)
summary(subset$APD40)
sd(subset$APD40)
summary(subset$APD50)
sd(subset$APD50)
summary(subset$APD60)
sd(subset$APD60)
summary(subset$APD70)
sd(subset$APD70)
summary(subset$APD80)
sd(subset$APD80)
summary(subset$APD90)
sd(subset$APD90)
summary(subset$Period.ms)
sd(subset$Period.ms, na.rm=TRUE)
mean(subset$Days.of.diff, na.rm=TRUE)
median(subset$Days.of.diff, na.rm=TRUE)
sd(subset$Days.of.diff, na.rm=TRUE)

subset2 <- subset[,c(4,5)]
subset2 %>% group_by(Cell.line) %>% count(Diff.protocol)


## Descriptive statistics per cell line ##

Line.1 <- filter(subset, Cell.line == 'Line 1')
summary(Line.1$MDP)
sd(Line.1$MDP)
summary(Line.1$APA)
sd(Line.1$APA)
summary(Line.1$Max.dV.dt)
sd(Line.1$Max.dV.dt)
summary(Line.1$APD90)
sd(Line.1$APD90)
summary(Line.1$Period.ms)
sd(Line.1$Period.ms, na.rm=TRUE)

Line.2 <- filter(subset, Cell.line == 'Line 2')
summary(Line.2$MDP)
sd(Line.2$MDP)
summary(Line.2$APA)
sd(Line.2$APA)
summary(Line.2$Max.dV.dt)
sd(Line.2$Max.dV.dt)
summary(Line.2$APD90)
sd(Line.2$APD90)
summary(Line.2$Period.ms)
```

```
sd(Line.2$Period.ms, na.rm=TRUE)

Line.3 <- filter(subset, Cell.line == 'Line 3')
summary(Line.3$MDP)
sd(Line.3$MDP)
summary(Line.3$APA)
sd(Line.3$APA)
summary(Line.3$Max.dV.dt)
sd(Line.3$Max.dV.dt)
summary(Line.3$APD90)
sd(Line.3$APD90)
summary(Line.3$Period.ms)
sd(Line.3$Period.ms, na.rm=TRUE)

Line.4 <- filter(subset, Cell.line == 'Line 4')
summary(Line.4$MDP)
sd(Line.4$MDP)
summary(Line.4$APA)
sd(Line.4$APA)
summary(Line.4$Max.dV.dt)
sd(Line.4$Max.dV.dt)
summary(Line.4$APD90)
sd(Line.4$APD90)
summary(Line.4$Period.ms)
sd(Line.4$Period.ms, na.rm=TRUE)

Line.5 <- filter(subset, Cell.line == 'Line 5')
summary(Line.5$MDP)
sd(Line.5$MDP)
summary(Line.5$APA)
sd(Line.5$APA)
summary(Line.5$Max.dV.dt)
sd(Line.5$Max.dV.dt)
summary(Line.5$APD90)
sd(Line.5$APD90)
summary(Line.5$Period.ms)
sd(Line.5$Period.ms, na.rm=TRUE)

Line.6 <- filter(subset, Cell.line == 'Line 6')
summary(Line.6$MDP)
sd(Line.6$MDP)
summary(Line.6$APA)
sd(Line.6$APA)
summary(Line.6$Max.dV.dt)
sd(Line.6$Max.dV.dt)
summary(Line.6$APD90)
sd(Line.6$APD90)
summary(Line.6$Period.ms)
sd(Line.6$Period.ms, na.rm=TRUE)


## FIGURE 1 - Density ##

p1 <- ggplot(aes(x = MDP), data = subset) + geom_density(size = 1) +
```

```
  scale_x_continuous(breaks = (seq(-100, -30, 10)), limits = c(-100,
-30)) + theme_bw(base_size = 20) +
  labs(title = '', x = 'Maximum diastolic potential (mV)', y =
'Density', tag = 'a') +
  theme(plot.margin = unit(c(0, 1, 0, 0), "cm"))
p2 <- ggplot(aes(x = APA), data = subset) + geom_density(size = 1) +
  scale_x_continuous(limits = c(40, 160), breaks = seq(40, 160, 20))
+ theme_bw(base_size = 20) +
  labs(title = '', x = 'Action potential amplitude (mV)', y =
'Density', tag = 'b') +
  theme(plot.margin = unit(c(0, 1, 0, 0), "cm"))
p3 <- ggplot(aes(x = Max.dV.dt), data = subset) + geom_density(size
= 1) +
  scale_x_log10(limits = c(1e3, 1e6), breaks = 10^seq(3,6),
labels=trans_format('log10',math_format(10^.x))) +
  theme_bw(base_size = 20) +
  labs(title = '', x = 'Maximum dV/dt (mV/s)', y = 'Density', tag =
'c') + theme(plot.margin = unit(c(0, 1, 0, 0), "cm"))
p4 <- ggplot(aes(x = APD90), data = subset) + geom_density(size = 1)
+
  scale_x_continuous(limits = c(0, 1000), breaks = seq(0, 1000,
100)) +
  theme_bw(base_size = 20) +
  labs(title = '', x = 'Action potential duration at 90% (ms)', y =
'Density', tag = 'd') +
  theme(plot.margin = unit(c(0, 1, 0, 0), "cm"))
p5 <- ggplot(aes(x = Period.ms), data = subset) + geom_density(size
= 1) +
  scale_x_continuous(limits = c(0, 5000), breaks = seq(0, 5000,
1000)) +
  theme_bw(base_size = 20) +
  labs(title = '', x = 'Cycle length (ms)', y = 'Density', tag =
'e') + theme(plot.margin = unit(c(0, 1, 0, 0), "cm"))

jpeg(filename="Figure 1.jpg", width=9, height=27, units='in',
res=600)
grid.arrange(p1, p2, p3, p4, p5, ncol = 1)
dev.off()


## FIGURE 2 - Cell type ##

p6 <- ggplot(aes(x = Cell.type, y = MDP), data = subset) +
geom_boxplot() +
  scale_y_continuous(breaks = (seq(-100, -30, 10)), limits = c(-100,
-30)) + coord_flip() + theme_bw(base_size = 20) +
  labs(title = '', x = '', y = 'Maximum diastolic potential (mV)',
tag = 'a') +
  theme(plot.margin = unit(c(0, 1, 0, 0), "cm"))
p7 <- ggplot(aes(x = Cell.type, y = APA), data = subset) +
geom_boxplot() +
  scale_y_continuous(limits = c(40, 160), breaks = seq(40, 160, 20))
+ coord_flip() + theme_bw(base_size = 20) +
  labs(title = '', x = '', y = 'Action potential amplitude (mV)',
```

```
tag = 'b') + theme(plot.margin = unit(c(0, 1, 0, 0), "cm")) +
  annotate('text', x = 1, y = 140, size = 6, label = '#', color =
'red')
p8 <- ggplot(aes(x = Cell.type, y = Max.dV.dt), data = subset) +
geom_boxplot() +
  scale_y_log10(limits = c(1e3, 1e6), breaks = 10^seq(3,6),
labels=trans_format('log10',math_format(10^.x))) +
  coord_flip() + theme_bw(base_size = 20) +
  labs(title = '', x = '', y = 'Maximum dV/dt (mV/s)', tag = 'c') +
theme(plot.margin = unit(c(0, 1, 0, 0), "cm")) +
  annotate('text', x = 1, y = 500000, size = 6, label = '#', color =
'red')
p9 <- ggplot(aes(x = Cell.type, y = APD90), data = subset) +
geom_boxplot() +
  scale_y_continuous(limits = c(0, 1000), breaks = seq(0, 1000,
100)) +
  coord_flip() + theme_bw(base_size = 20) +
  labs(title = '', x = '', y = 'Action potential duration at 90%
(ms)', tag = 'd') + theme(plot.margin = unit(c(0, 1, 0, 0), "cm")) +
  annotate('text', x = 1, y = 950, size = 6, label = '#', color =
'red')
p10 <- ggplot(aes(x = Cell.type, y = Period.ms), data = subset) +
geom_boxplot() +
  scale_y_continuous(limits = c(0, 5000), breaks = seq(0, 5000,
1000)) +
  coord_flip() + theme_bw(base_size = 20) +
  labs(title = '', x = '', y = 'Cycle length (ms)', tag = 'e') +
theme(plot.margin = unit(c(0, 1, 0, 0), "cm")) +
  annotate('text', x = 1, y = 4500, size = 6, label = '#', color =
'red')

jpeg(filename="Figure 2.jpg", width=9, height=27, units='in',
res=600)
grid.arrange(p6, p7, p8, p9, p10, ncol = 1)
dev.off()


## FIGURE 3 - Cell line ##

p11 <- ggplot(aes(x = Cell.line, y = MDP), data = subset) +
geom_boxplot() +
  scale_y_continuous(breaks = (seq(-100, -30, 10)), limits = c(-100,
-30)) + coord_flip() + theme_bw(base_size = 20) +
  labs(title = '', x = '', y = 'Maximum diastolic potential (mV)',
tag = 'a') + theme(plot.margin = unit(c(0, 1, 0, 0), "cm")) +
  annotate('text', x = 1, y = -35, size = 6, label = '#', color =
'red') +
  annotate('text', x = 4, y = -35, size = 6, label = '#', color =
'orange')
p12 <- ggplot(aes(x = Cell.line, y = APA), data = subset) +
geom_boxplot() +
  scale_y_continuous(limits = c(40, 160), breaks = seq(40, 160, 20))
+ coord_flip() + theme_bw(base_size = 20) +
  labs(title = '', x = '', y = 'Action potential amplitude (mV)',
```

```
tag = 'b') + theme(plot.margin = unit(c(0, 1, 0, 0), "cm")) +
  annotate('text', x = 1, y = 140, size = 6, label = '#', color =
'red') +
  annotate('text', x = 3, y = 140, size = 6, label = '#', color =
'green') +
  annotate('text', x = 5, y = 140, size = 6, label = '#', color =
'pink')
p13 <- ggplot(aes(x = Cell.line, y = Max.dV.dt), data = subset) +
geom_boxplot() +
  scale_y_log10(limits = c(1e3, 1e6), breaks = 10^seq(3,6),
labels=trans_format('log10',math_format(10^.x))) +
  coord_flip() + theme_bw(base_size = 20) +
  labs(title = '', x = '', y = 'Maximum dV/dt (mV/s)', tag = 'c') +
theme(plot.margin = unit(c(0, 1, 0, 0), "cm")) +
  annotate('text', x = 1, y = 500000, size = 6, label = '#', color =
'red') +
  annotate('text', x = 3, y = 500000, size = 6, label = '#', color =
'green') +
  annotate('text', x = 6, y = 500000, size = 6, label = '#', color =
'purple')
p14 <- ggplot(aes(x = Cell.line, y = APD90), data = subset) +
geom_boxplot() +
  scale_y_continuous(limits = c(0, 1000), breaks = seq(0, 1000,
100)) +
  coord_flip() + theme_bw(base_size = 20) +
  labs(title = '', x = '', y = 'Action potential duration at 90%
(ms)', tag = 'd') + theme(plot.margin = unit(c(0, 0, 0, 0), "cm")) +
  annotate('text', x = 1, y = 950, size = 6, label = '#', color =
'red') +
  annotate('text', x = 2, y = 950, size = 6, label = '#', color =
'blue') +
  annotate('text', x = 3, y = 950, size = 6, label = '#', color =
'green') +
  annotate('text', x = 4, y = 950, size = 6, label = '#', color =
'orange') +
  annotate('text', x = 5, y = 950, size = 6, label = '#', color =
'pink')
p15 <- ggplot(aes(x = Cell.line, y = Period.ms), data = subset) +
geom_boxplot() +
  scale_y_continuous(limits = c(0, 5000), breaks = seq(0, 5000,
1000)) +
  coord_flip() + theme_bw(base_size = 20) +
  labs(title = '', x = '', y = 'Cycle length (ms)', tag = 'e') +
theme(plot.margin = unit(c(0, 0, 0, 0), "cm")) +
  annotate('text', x = 1, y = 5000, size = 6, label = '#', color =
'red') +
  annotate('text', x = 2, y = 5000, size = 6, label = '#', color =
'blue') +
  annotate('text', x = 3, y = 5000, size = 6, label = '#', color =
'green') +
  annotate('text', x = 4, y = 5000, size = 6, label = '#', color =
'orange') +
  annotate('text', x = 5, y = 5000, size = 6, label = '#', color =
'pink')
```

```
jpeg(filename="Figure 3.jpg", width=9, height=27, units='in',
res=600)
grid.arrange(p11, p12, p13, p14, p15, ncol = 1)
dev.off()


# Statistics for figures 1, 2 and 3
t.test(subset$MDP~subset$Cell.type)
anovaMDPCL <- aov(MDP ~ Cell.line, data = subset)
Anova(anovaMDPCL, type = 3)
TukeyHSD(anovaMDPCL)
kruskal.test(MDP ~ Cell.line, data = subset)

t.test(subset$APA~subset$Cell.type)
anovaAPACL <- aov(APA ~ Cell.line, data = subset)
Anova(anovaAPACL, type = 3)
TukeyHSD(anovaAPACL)
kruskal.test(APA ~ Cell.line, data = subset)

t.test(subset$Max.dV.dt~subset$Cell.type)
anovaMax.dV.dtCL <- aov(Max.dV.dt ~ Cell.line, data = subset)
Anova(anovaMax.dV.dtCL, type = 3)
TukeyHSD(anovaMax.dV.dtCL)
kruskal.test(Max.dV.dt ~ Cell.line, data = subset)

t.test(subset$APD90~subset$Cell.type)
anovaAPD90CL <- aov(APD90 ~ Cell.line, data = subset)
Anova(anovaAPD90CL, type = 3)
TukeyHSD(anovaAPD90CL)
kruskal.test(APD90 ~ Cell.line, data = subset)

t.test(subset$Period.ms~subset$Cell.type)
anovaPeriod.msCL <- aov(Period.ms ~ Cell.line, data = subset)
Anova(anovaPeriod.msCL, type = 3)
TukeyHSD(anovaPeriod.msCL)
kruskal.test(Period.ms ~ Cell.line, data = subset)


## FIGURE 4 - Classic physiologic correlations for the entire
dataset ##

# MDP vs APA
cor1 <- cor.test(subset$MDP, subset$APA)$estimate
cor2 <- cor.test(subset$MDP, subset$APA)$p.value
p16 <- ggplot(aes(x = MDP, y = APA),
              data = subset) +
  geom_point() +
  xlim(-100, -20) + ylim(50, 150) +
  labs(title = '', x = 'Maximum diastolic potential (mV)',
       y = 'Action potential amplitude (mV)', tag = 'a') +
  theme(plot.title = element_text(size = 11)) + theme_bw() +
  annotate('text', x = -35, y = 150, size = 5, label = sprintf('r =
%0.3f', cor1)) +
```

```
  annotate('text', x = -35, y = 140, size = 5, label = sprintf('p =
%0.3e', cor2))

# MDP vs Maximum dV/dt
cor3 <- cor.test(subset$MDP, subset$Max.dV.dt)$estimate
cor4 <- cor.test(subset$MDP, subset$Max.dV.dt)$p.value
p17 <- ggplot(aes(x = MDP, y = Max.dV.dt),
             data = subset) +
  geom_point() +
  xlim(-100, -20) + scale_y_log10(limits = c(1e3, 1e6), breaks =
10^seq(3,6),

labels=trans_format('log10',math_format(10^.x))) +
  labs(title = '', x = 'Maximum diastolic potential (mV)',
       y = 'Maximum dV/dt (mV/s)', tag = 'b') +
  theme(plot.title = element_text(size = 11)) + theme_bw() +
  annotate('text', x = -35, y = 1e6, size = 5, label = sprintf('r =
%0.3f', cor3)) +
  annotate('text', x = -35, y = 600000, size = 5, label = sprintf('p
= %0.3e', cor4))

# APD90 vs Period
cor5 <- cor.test(subset$APD90, subset$Period.ms)$estimate
cor6 <- cor.test(subset$APD90, subset$Period.ms)$p.value
p18 <- ggplot(aes(x = Period.ms, y = APD90),
             data = subset) +
  geom_point() +
  xlim(0, 5000) + ylim(0, 1000) +
  labs(title = '', x = 'Cycle length (ms)',
       y = 'Action potential duration at 90% (ms)', tag = 'c') +
  theme(plot.title = element_text(size = 11)) + theme_bw() +
  annotate('text', x = 4000, y = 1000, size = 5, label = sprintf('r
= %0.3f', cor5)) +
  annotate('text', x = 4000, y = 930, size = 5, label = sprintf('p =
%0.3e', cor6))

jpeg(filename="Figure 4.jpg", width=12, height=4, units='in',
res=600)
grid.arrange(p16, p17, p18, ncol = 3)
dev.off()


## SUPPL FIGURE 1 - Classic physiologic correlations for subsets ##

p19 <- ggplot(aes(x = MDP, y = APA, color = Cell.type),
              data = subset) +
  geom_point() +
  xlim(-100, -20) + ylim(50, 150) +
  labs(title = '', x = 'Maximum diastolic potential (mV)',
       y = 'Action potential amplitude (mV)', tag = 'a', colour =
'Cell type') +
  theme(plot.title = element_text(size = 11)) + theme_bw()

p20 <- ggplot(aes(x = MDP, y = APA, color = Diff.protocol),
```

```
              data = subset) +
  geom_point() +
  xlim(-100, -20) + ylim(50, 150) +
  labs(title = '', x = 'Maximum diastolic potential (mV)',
       y = 'Action potential amplitude (mV)', tag = 'b', colour =
c('Differentiation\nprotocol')) +
  theme(plot.title = element_text(size = 11)) + theme_bw()

p21 <- ggplot(aes(x = MDP, y = APA, color = Cell.line),
              data = subset) +
  geom_point() +
  xlim(-100, -20) + ylim(50, 150) +
  labs(title = '', x = 'Maximum diastolic potential (mV)',
       y = 'Action potential amplitude (mV)', tag = 'c', colour =
'Cell line') +
  theme(plot.title = element_text(size = 11)) + theme_bw()

p22 <- ggplot(aes(x = MDP, y = Max.dV.dt, color = Cell.type),
              data = subset) +
  geom_point() +
  xlim(-100, -20) + scale_y_log10(limits = c(1e3, 1e6), breaks =
10^seq(3,6),

labels=trans_format('log10',math_format(10^.x))) +
  labs(title = '', x = 'Maximum diastolic potential (mV)',
       y = 'Maximum dV/dt (mV/s)', tag = 'd', colour = 'Cell type')
+
  theme(plot.title = element_text(size = 11)) + theme_bw()
p23 <- ggplot(aes(x = MDP, y = Max.dV.dt, color = Diff.protocol),
              data = subset) +
  geom_point() +
  xlim(-100, -20) + scale_y_log10(limits = c(1e3, 1e6), breaks =
10^seq(3,6),

labels=trans_format('log10',math_format(10^.x))) +
  labs(title = '', x = 'Maximum diastolic potential (mV)',
       y = 'Maximum dV/dt (mV/s)', tag = 'e', colour =
c('Differentiation\nprotocol')) +
  theme(plot.title = element_text(size = 11)) + theme_bw()
p24 <- ggplot(aes(x = MDP, y = Max.dV.dt, color = Cell.line),
              data = subset) +
  geom_point() +
  xlim(-100, -20) + scale_y_log10(limits = c(1e3, 1e6), breaks =
10^seq(3,6),

labels=trans_format('log10',math_format(10^.x))) +
  labs(title = '', x = 'Maximum diastolic potential (mV)',
       y = 'Maximum dV/dt (mV/s)', tag = 'f', colour = 'Cell line')
+
  theme(plot.title = element_text(size = 11)) + theme_bw()

p25 <- ggplot(aes(x = Period.ms, y = APD90, color = Cell.type),
              data = subset) +
  geom_point() +
```

```
  xlim(0, 5000) + ylim(0, 1000) +
  labs(title = '', x = 'Cycle length (ms)',
       y = 'Action potential duration at 90% (ms)', tag = 'g',
colour = 'Cell type') +
  theme(plot.title = element_text(size = 11)) + theme_bw()
p26 <- ggplot(aes(x = Period.ms, y = APD90, color = Diff.protocol),
              data = subset) +
  geom_point() +
  xlim(0, 5000) + ylim(0, 1000) +
  labs(title = '', x = 'Cycle length (ms)',
       y = 'Action potential duration at 90% (ms)', tag = 'h',
colour = c('Differentiation\nprotocol')) +
  theme(plot.title = element_text(size = 11)) + theme_bw()
p27 <- ggplot(aes(x = Period.ms, y = APD90, color = Cell.line),
              data = subset) +
  geom_point() +
  xlim(0, 5000) + ylim(0, 1000) +
  labs(title = '', x = 'Cycle length (ms)',
       y = 'Action potential duration at 90% (ms)', tag = 'i',
colour = 'Cell line') +
  theme(plot.title = element_text(size = 11)) + theme_bw()

jpeg(filename="Suppl Fig 1.jpg", width=16, height=12, units='in',
res=600)
grid.arrange(p19, p20, p21, p22, p23, p24, p25, p26, p27, ncol = 3)
dev.off()


## FIGURE 5 - Influence of differentiation batch ##

Line.1.prot.1 <- filter(Line.1, Diff.protocol == 'Protocol 1')
Line.2.prot.2 <- filter(Line.2, Diff.protocol == 'Protocol 2')
Line.4.prot.2 <- filter(Line.4, Diff.protocol == 'Protocol 2')

p28 <- ggplot(aes(x = Diff.ID, y = MDP), data = Line.1.prot.1) +
geom_boxplot() +
  scale_y_continuous(breaks = (seq(-100, -20, 20)), limits = c(-100,
-20)) + theme_bw(base_size = 16) +
  labs(title = 'Line 1', x = '', y = 'MDP (mV)', tag = 'a') +
  theme(plot.margin = unit(c(0, 0, 0, 0), "cm"), axis.text.x =
element_text(angle = 45, hjust = 1))
p29 <- ggplot(aes(x = Diff.ID, y = MDP), data = Line.2.prot.2) +
geom_boxplot() +
  scale_y_continuous(breaks = (seq(-100, -20, 20)), limits = c(-100,
-20)) + theme_bw(base_size = 16) +
  labs(title = 'Line 2', x = '', y = 'MDP (mV)', tag = 'b') +
  theme(plot.margin = unit(c(0, 0, 0, 0), "cm"), axis.text.x =
element_text(angle = 45, hjust = 1))
p30 <- ggplot(aes(x = Diff.ID, y = MDP), data = Line.4.prot.2) +
geom_boxplot() +
  scale_y_continuous(breaks = (seq(-100, -20, 20)), limits = c(-100,
-20)) + theme_bw(base_size = 16) +
  labs(title = 'Line 4', x = '', y = 'MDP (mV)', tag = 'c') +
  theme(plot.margin = unit(c(0, 0, 0, 0), "cm"), axis.text.x =
```

```
element_text(angle = 45, hjust = 1))

p31 <- ggplot(aes(x = Diff.ID, y = APA), data = Line.1.prot.1) +
geom_boxplot() +
  scale_y_continuous(limits = c(0, 150), breaks = seq(0, 150, 20)) +
theme_bw(base_size = 16) +
  labs(title = 'Line 1', x = '', y = 'APA (mV)', tag = 'd') +
  theme(plot.margin = unit(c(0, 0, 0, 0), "cm"), axis.text.x =
element_text(angle = 45, hjust = 1))
p32 <- ggplot(aes(x = Diff.ID, y = APA), data = Line.2.prot.2) +
geom_boxplot() +
  scale_y_continuous(limits = c(0, 150), breaks = seq(0, 150, 20)) +
theme_bw(base_size = 16) +
  labs(title = 'Line 2', x = '', y = 'APA (mV)', tag = 'e') +
  theme(plot.margin = unit(c(0, 0, 0, 0), "cm"), axis.text.x =
element_text(angle = 45, hjust = 1))
p33 <- ggplot(aes(x = Diff.ID, y = APA), data = Line.4.prot.2) +
geom_boxplot() +
  scale_y_continuous(limits = c(0, 150), breaks = seq(0, 150, 20)) +
theme_bw(base_size = 16) +
  labs(title = 'Line 4', x = '', y = 'APA (mV)', tag = 'f') +
  theme(plot.margin = unit(c(0, 0, 0, 0), "cm"), axis.text.x =
element_text(angle = 45, hjust = 1))

p34 <- ggplot(aes(x = Diff.ID, y = Max.dV.dt), data = Line.1.prot.1)
+ geom_boxplot() +
  scale_y_log10(limits = c(1e3, 1e6), breaks = 10^seq(3,6),
labels=trans_format('log10',math_format(10^.x))) +
  theme_bw(base_size = 16) + labs(title = 'Line 1', x = '', y = 'Max
dV/dt (mV/s)', tag = 'g') +
  theme(plot.margin = unit(c(0, 0, 0, 0), "cm"), axis.text.x =
element_text(angle = 45, hjust = 1))
p35 <- ggplot(aes(x = Diff.ID, y = Max.dV.dt), data = Line.2.prot.2)
+ geom_boxplot() +
  scale_y_log10(limits = c(1e3, 1e6), breaks = 10^seq(3,6),
labels=trans_format('log10',math_format(10^.x))) +
  theme_bw(base_size = 16) + labs(title = 'Line 2', x = '', y = 'Max
dV/dt (mV/s)', tag = 'h') +
  theme(plot.margin = unit(c(0, 0, 0, 0), "cm"), axis.text.x =
element_text(angle = 45, hjust = 1))
p36 <- ggplot(aes(x = Diff.ID, y = Max.dV.dt), data = Line.4.prot.2)
+ geom_boxplot() +
  scale_y_log10(limits = c(1e3, 1e6), breaks = 10^seq(3,6),
labels=trans_format('log10',math_format(10^.x))) +
  theme_bw(base_size = 16) + labs(title = 'Line 4', x = '', y = 'Max
dV/dt (mV/s)', tag = 'i') +
  theme(plot.margin = unit(c(0, 0, 0, 0), "cm"), axis.text.x =
element_text(angle = 45, hjust = 1))

p37 <- ggplot(aes(x = Diff.ID, y = APD90), data = Line.1.prot.1) +
geom_boxplot() +
  scale_y_continuous(limits = c(0, 600), breaks = seq(0, 600, 100))
+
  theme_bw(base_size = 16) + labs(title = 'Line 1', x = '', y =
```

```
'APD90 (ms)', tag = 'j') +
  theme(plot.margin = unit(c(0, 0, 0, 0), "cm"), axis.text.x =
element_text(angle = 45, hjust = 1))
p38 <- ggplot(aes(x = Diff.ID, y = APD90), data = Line.2.prot.2) +
geom_boxplot() +
  scale_y_continuous(limits = c(0, 600), breaks = seq(0, 600, 100))
+
  theme_bw(base_size = 16) + labs(title = 'Line 2', x = '', y =
'APD90 (ms)', tag = 'k') +
  theme(plot.margin = unit(c(0, 0, 0, 0), "cm"), axis.text.x =
element_text(angle = 45, hjust = 1))
p39 <- ggplot(aes(x = Diff.ID, y = APD90), data = Line.4.prot.2) +
geom_boxplot() +
  scale_y_continuous(limits = c(0, 600), breaks = seq(0, 600, 100))
+
  theme_bw(base_size = 16) + labs(title = 'Line 4', x = '', y =
'APD90 (ms)', tag = 'l') +
  theme(plot.margin = unit(c(0, 0, 0, 0), "cm"), axis.text.x =
element_text(angle = 45, hjust = 1))

p40 <- ggplot(aes(x = Diff.ID, y = Period.ms), data = Line.1.prot.1)
+ geom_boxplot() +
  scale_y_continuous(limits = c(0, 4000), breaks = seq(0, 4000,
1000)) +
  theme_bw(base_size = 16) + labs(title = 'Line 1', x = '', y =
'Cycle length (ms)', tag = 'm') +
  theme(plot.margin = unit(c(0, 0, 0, 0), "cm"), axis.text.x =
element_text(angle = 45, hjust = 1))
p41 <- ggplot(aes(x = Diff.ID, y = Period.ms), data = Line.2.prot.2)
+ geom_boxplot() +
  scale_y_continuous(limits = c(0, 4000), breaks = seq(0, 4000,
1000)) +
  theme_bw(base_size = 16) + labs(title = 'Line 2', x = '', y =
'Cycle length (ms)', tag = 'n') +
  theme(plot.margin = unit(c(0, 0, 0, 0), "cm"), axis.text.x =
element_text(angle = 45, hjust = 1))
p42 <- ggplot(aes(x = Diff.ID, y = Period.ms), data = Line.4.prot.2)
+ geom_boxplot() +
  scale_y_continuous(limits = c(0, 4000), breaks = seq(0, 4000,
1000)) +
  theme_bw(base_size = 16) + labs(title = 'Line 4', x = '', y =
'Cycle length (ms)', tag = 'o') +
  theme(plot.margin = unit(c(0, 0, 0, 0), "cm"), axis.text.x =
element_text(angle = 45, hjust = 1))

jpeg(filename="Figure 5.jpg", width=18, height=24, units='in',
res=600)
grid.arrange(p28, p29, p30, p31, p32, p33, p34, p35, p36, p37, p38,
p39, p40, p41, p42, ncol = 3)
dev.off()


# FIGURE 6 - Influence of differentiation protocol ##
```

```
p43 <- ggplot(aes(x = Diff.protocol, y = MDP), data = Line.2) +
geom_boxplot() +
  scale_y_continuous(breaks = seq(-100, 0, 20), limits = c(-100,
-20)) + theme_bw(base_size = 16) +
  labs(title = '', x = '', y = 'Maximum diastolic potential (mV)',
tag = 'a') +
  theme(plot.margin = unit(c(0, 1, 0, 0), "cm"))

t.test(Line.2$MDP~Line.2$Diff.protocol)

p44 <- ggplot(aes(x = Diff.protocol, y = APA), data = Line.2) +
geom_boxplot() +
  scale_y_continuous(limits = c(0, 140), breaks = seq(0, 140, 20)) +
theme_bw(base_size = 16) +
  labs(title = '', x = '', y = 'Action potential amplitude (mV)',
tag = 'b') +
  theme(plot.margin = unit(c(0, 1, 0, 0), "cm")) +
  annotate('text', x = 2, y = 120, size = 5, label = '#', color =
'red')

t.test(Line.2$APA~Line.2$Diff.protocol)

p45 <- ggplot(aes(x = Diff.protocol, y = Max.dV.dt), data = Line.2)
+ geom_boxplot() +
  scale_y_log10(limits = c(1e3, 1e6), breaks = 10^seq(3,6),
labels=trans_format('log10',math_format(10^.x))) +
  theme_bw(base_size = 16) + labs(title = '', x = '', y = 'Maximum
dV/dt (mV/s)', tag = 'c') +
  theme(plot.margin = unit(c(0, 1, 0, 0), "cm")) +
  annotate('text', x = 2, y = 500000, size = 5, label = '#', color =
'red')

t.test(Line.2$Max.dV.dt~Line.2$Diff.protocol)

p46 <- ggplot(aes(x = Diff.protocol, y = APD90), data = Line.2) +
geom_boxplot() +
  scale_y_continuous(limits = c(0, 600), breaks = seq(0, 600, 100))
+
  theme_bw(base_size = 16) + labs(title = '', x = '', y = 'Action
potential duration at 90% (ms)', tag = 'd') +
  theme(plot.margin = unit(c(0, 1, 0, 0), "cm")) +
  annotate('text', x = 2, y = 500, size = 5, label = '#', color =
'red')

t.test(Line.2$APD90~Line.2$Diff.protocol)

p47 <- ggplot(aes(x = Diff.protocol, y = Period.ms), data = Line.2)
+ geom_boxplot() +
  scale_y_continuous(limits = c(0, 5000), breaks = seq(0, 5000,
1000)) +
  theme_bw(base_size = 16) + labs(title = '', x = '', y = 'Cycle
length (ms)', tag = 'e') +
  theme(plot.margin = unit(c(0, 1, 0, 0), "cm"))
```

```
t.test(Line.2$Period.ms~Line.2$Diff.protocol)

jpeg(filename="Figure 6.jpg", width=12, height=12, units='in',
res=600)
grid.arrange(p43, p44, p45, p46, p47, ncol = 2)
dev.off()


## FIGURE 7 - Effect of pacing ##

# Considering the entire dataset

Line.2.4.prot.2 <- filter(subset, Diff.protocol == 'Protocol 2') %>%
  subset(Cell.line == 'Line 2' | Cell.line == 'Line 4')

p48 <- ggplot(aes(x = Beating, y = APD90), data = Line.2.4.prot.2) +
geom_boxplot() +
  scale_x_discrete(labels = c('Pacing','Spontaneous')) +
  scale_y_continuous(limits = c(0, 800), breaks = seq(0, 800, 100))
+
  theme_bw(base_size = 16) + labs(title = 'All cells', x = '', y =
'APD90 (ms)', tag = 'a') +
  theme(plot.margin = unit(c(0, 1, 0, 0), "cm")) +
  annotate('text', x = 2, y = 800, size = 5, label = '#', color =
'red')

t.test(Line.2.4.prot.2$APD90~Line.2.4.prot.2$Beating)

subset3 <- subset[,c(4,5,9)]
subset3 %>% group_by(Cell.line, Diff.protocol) %>% count(Beating)

# Considering similar cycle lengths
Line.2.4.prot.2.period <- filter(Line.2.4.prot.2, Period.ms > 900,
Period.ms < 1100)

p49 <- ggplot(aes(x = Beating, y = APD90), data =
Line.2.4.prot.2.period) + geom_boxplot() +
  scale_x_discrete(labels = c('Pacing','Spontaneous')) +
  scale_y_continuous(limits = c(0, 600), breaks = seq(0, 600, 100))
+
  theme_bw(base_size = 16) + labs(title = '1100 > Spontaneous cycle
length > 900', x = '', y = 'APD90 (ms)', tag = 'b') +
  theme(plot.margin = unit(c(0, 1, 0, 0), "cm"))

t.test(Line.2.4.prot.2.period$APD90~Line.2.4.prot.2.period$Beating)

subset4 <- Line.2.4.prot.2.period[,c(4,5,9)]
subset4 %>% group_by(Cell.line, Diff.protocol) %>% count(Beating)

jpeg(filename="Figure 7.jpg", width=12, height=6, units='in',
res=600)
grid.arrange(p48, p49, ncol = 2)
dev.off()
```

```
## FIGURE 8 - Effect of differentiation time ##

subset.group.days <- subset %>% drop_na(Group.days) %>%
filter(Diff.protocol == 'Protocol 2') %>%
  subset(Cell.line == 'Line 2' | Cell.line == 'Line 4')

data.frame(table(subset.group.days$Group.days))
subset.group.days %>% group_by(Group.days) %>%
summarise_at(vars(APD90), list(name = median, mean, sd))

p50 <- ggplot(aes(x = Group.days, y = MDP), data =
subset.group.days) + geom_boxplot() +
  scale_x_discrete(labels = c('< 30','> 30')) +
  scale_y_continuous(breaks = seq(-100, 0, 20), limits = c(-100,
-20)) + theme_bw(base_size = 16) +
  labs(title = '', x = 'Days of differentiation', y = 'Maximum
diastolic potential (mV)', tag = 'a') +
  theme(plot.margin = unit(c(0, 1, 0, 0), "cm"))
p51 <- ggplot(aes(x = Group.days, y = APA), data =
subset.group.days) + geom_boxplot() +
  scale_x_discrete(labels = c('< 30','> 30')) +
  scale_y_continuous(limits = c(0, 140), breaks = seq(0, 140, 20)) +
theme_bw(base_size = 16) +
  labs(title = '', x = 'Days of differentiation', y = 'Action
potential amplitude (mV)', tag = 'b') +
  theme(plot.margin = unit(c(0, 1, 0, 0), "cm")) +
  annotate('text', x = 2, y = 135, size = 5, label = '#', color =
'red')
p52 <- ggplot(aes(x = Group.days, y = Max.dV.dt), data =
subset.group.days) + geom_boxplot() +
  scale_x_discrete(labels = c('< 30','> 30')) +
  scale_y_log10(limits = c(1e3, 1e6), breaks = 10^seq(3,6),
labels=trans_format('log10',math_format(10^.x))) +
  theme_bw(base_size = 16) + labs(title = '', x = 'Days of
differentation', y = 'Maximum dV/dt (mV/s)', tag = 'c') +
  theme(plot.margin = unit(c(0, 1, 0, 0), "cm")) +
  annotate('text', x = 2, y = 500000, size = 5, label = '#', color =
'red')
p53 <- ggplot(aes(x = Group.days, y = APD90), data =
subset.group.days) + geom_boxplot() +
  scale_x_discrete(labels = c('< 30','> 30')) +
  scale_y_continuous(limits = c(0, 700), breaks = seq(0, 700, 100))
+
  theme_bw(base_size = 16) + labs(title = , x = 'Days of
differentiation', y = 'APD90 (ms)', tag = 'd') +
  theme(plot.margin = unit(c(0, 1, 0, 0), "cm")) +
  annotate('text', x = 2, y = 700, size = 5, label = '#', color =
'red')
p54 <- ggplot(aes(x = Group.days, y = Period.ms), data =
subset.group.days) + geom_boxplot() +
  scale_x_discrete(labels = c('< 30','> 30')) +
  scale_y_continuous(limits = c(0, 5000), breaks = seq(0, 5000,
1000)) +
```

```
  theme_bw(base_size = 16) + labs(title = '', x = 'Days of
differentation', y = 'Cycle length (ms)', tag = 'e') +
  theme(plot.margin = unit(c(0, 1, 0, 0), "cm"))

t.test(subset.group.days$MDP~subset.group.days$Group.days)
t.test(subset.group.days$APA~subset.group.days$Group.days)
t.test(subset.group.days$Max.dV.dt~subset.group.days$Group.days)
t.test(subset.group.days$APD90~subset.group.days$Group.days)
t.test(subset.group.days$Period.ms~subset.group.days$Group.days)

jpeg(filename="Figure 8.jpg", width=12, height=12, units='in',
res=600)
grid.arrange(p50, p51, p52, p53, p54, ncol = 2)
dev.off()

#################################
             #END#
#################################
```
