# Supplementary material for: Action potential variability in human pluripotent stem cell-derived cardiomyocytes obtained from healthy donors
Source: Front Physiol. 2022 Dec 16;13:1077069. doi: 10.3389/fphys.2022.1077069 (PMC9800870; doi:10.3389/fphys.2022.1077069)
Supplement: Supplementary file 2 [file Table1.DOCX]

Suppl Table 1: Characteristics of cell line donors

| **Cell line code** | **Source** | **Sex** | **Race** | **Age (years)** | **Reference** |
| --- | --- | --- | --- | --- | --- |
| Line 1 | ES | Female | Not provided | Not applicable | Elliot et al., 2011 |
| Line 2 | iPS | Male | White | 27 | Mesquita et al., 2019 |
| Line 3 | iPS | Female | White | 13 | Kasai-Brunswick et al., 2018 |
| Line 4 | iPS | Male | Black | 64 | Kasai-Brunswick et al., 2018 |
| Line 5 | iPS | Female | White | 10 | Unpublished |
| Line 6 | iPS | Male | Not provided | 30-40 | Cruvinel et al., 2020 |

ES: embryonic stem cells, iPS: induced pluripotent stem cells.
