# Supplementary material for: Action potential variability in human pluripotent stem cell-derived cardiomyocytes obtained from healthy donors
Source: Front Physiol. 2022 Dec 16;13:1077069. doi: 10.3389/fphys.2022.1077069 (PMC9800870; doi:10.3389/fphys.2022.1077069)
Supplement: Supplementary file 3 [file Table8.DOCX]

Suppl Table 8: Descriptive statistics for cycle length in ms per cell line

|  | Line 1 | Line 2 | Line 3 | Line 4 | Line 5 | Line 6 |
| --- | --- | --- | --- | --- | --- | --- |
| Min | 394.4 | 309.7 | 880.0 | 491.3 | 460.0 | 768.7 |
| 1^st^ Q | 726.0 | 1125.2 | 1714.0 | 1101.2 | 1975.0 | 1260.0 |
| Median | 997.0 | 1634.0 | 2556.0 | 1424.5 | 2542.0 | 1658.0 |
| 3^rd^ Q | 1573.2 | 1891.2 | 2636.0 | 1953.0 | 3208.0 | 2099.0 |
| Max | 2657.0 | 4467.0 | 8634.0 | 4260.0 | 5533.0 | 3821.0 |
| Mean | 1134.9 | 1891.2 | 2636.0 | 1530.3 | 2633.0 | 1811.4 |
| SD | 551.5 | 708.7 | 1335.1 | 656.6 | 1152.5 | 824.9 |

Min: minimum, 1^st^ Q: first quartile, 3^rd^ Q: third quartile, Max: maximum, SD: standard deviation.
