# Supplementary material for: Action potential variability in human pluripotent stem cell-derived cardiomyocytes obtained from healthy donors
Source: Front Physiol. 2022 Dec 16;13:1077069. doi: 10.3389/fphys.2022.1077069 (PMC9800870; doi:10.3389/fphys.2022.1077069)
Supplement: Supplementary file 5 [file Table7.DOCX]

Suppl Table 7: Descriptive statistics for APD90 in ms per cell line

|  | Line 1 | Line 2 | Line 3 | Line 4 | Line 5 | Line 6 |
| --- | --- | --- | --- | --- | --- | --- |
| Min | 139.4 | 115.3 | 206.5 | 112.8 | 256.0 | 260.6 |
| 1^st^ Q | 176.9 | 205.4 | 319.2 | 176.0 | 294.1 | 417.3 |
| Median | 217.2 | 232.8 | 411.5 | 224.9 | 320.3 | 519.3 |
| 3^rd^ Q | 258.0 | 294.2 | 493.1 | 314.7 | 357.0 | 599.8 |
| Max | 697.3 | 556.1 | 1290.0 | 879.7 | 595.4 | 1269.0 |
| Mean | 235.8 | 253.0 | 453.0 | 264.6 | 348.7 | 546.2 |
| SD | 94.5 | 72.66 | 206.6 | 124.2 | 86.6 | 190.6 |

Min: minimum, 1^st^ Q: first quartile, 3^rd^ Q: third quartile, Max: maximum, SD: standard deviation.
