# Supplementary material for: Action potential variability in human pluripotent stem cell-derived cardiomyocytes obtained from healthy donors
Source: Front Physiol. 2022 Dec 16;13:1077069. doi: 10.3389/fphys.2022.1077069 (PMC9800870; doi:10.3389/fphys.2022.1077069)
Supplement: Supplementary file 6 [file Table9.DOCX]

Suppl Table 9: Standard deviation of electrophysiologic parameters in animals and PSC-derived cardiomyocytes

|  | | Standard deviation | | | |  | |
| --- | --- | --- | --- | --- | --- | --- | --- |
| Cell type | Species | RMP/MDP (mV) | APA (mV) | dV/dt max (V/s) | APD90 (ms) | n | Reference |
| Isolated ventricular myocytes | Rat | 10.29 | 19.02 | 99.06 | na | 20 | Howlett et al., 2022 |
| Isolated ventricular myocytes | Rat | na | 7.86 | na | 22.83 | 8 | Tan et al., 2014 |
| Isolated ventricular myocytes | Guinea pig | na | na | 11.00 | 11.60 | 30 | Al-Owais et al., 2021 |
| Ventricular myocytes (slice) | Guinea pig | 6.14 | 5.38 | 53.00 | 30.72 | 59 | Bussek et al., 2009 |
| Ventricular myocytes (slice) | Mouse | 4.38 | 6.02 | 19.72 | 8.76 | 30 | Halbach et al., 2006 |
| Ventricular myocytes (slice) | Mouse | 6.35 | na | na | 7.14 | 7 | Saito et al., 2004 |
| PSC-derived cardiomyocytes | Human | 9.26 | 11.53 | 18.00 | 136.74 (82.34*) | 780 (124*) | - |

*refers to a subset of paced cells. RMP: resting membrane potential, MDP: maximum diastolic potentials, APA: action potential amplitudes, dV/dt max: maximum dV/dt, APD90: action potential duration at 90% repolarization.
