# Supplementary material for: Action potential variability in human pluripotent stem cell-derived cardiomyocytes obtained from healthy donors
Source: Front Physiol. 2022 Dec 16;13:1077069. doi: 10.3389/fphys.2022.1077069 (PMC9800870; doi:10.3389/fphys.2022.1077069)
Supplement: Supplementary file 7 [file Table2.DOCX]

Suppl Table 2: Culture and differentiation conditions for each cell line

| Cell line | Culture | | Differentiation | |
| --- | --- | --- | --- | --- |
|  | Format | Medium | Format | Method |
| Line 1 | MEF | hES | Embryoid body | Kattman: StemPro34, Wnt activation with BMP4, Activin A and bFGF; Wnt inhibition with XAV939 |
| Line 2* | MEF | hES | Monolayer | Lian: RPMI+ B27, Wnt activation with CHIR99021; Wnt inhibition with XAV939 |
| Line 3 | MEF | hES | Monolayer |  |
| Line 4 | MEF | hES | Monolayer |  |
| Line 5 | MEF | hES | Monolayer |  |
| Line 6 | Feeder-free (Geltrex) | Essential 8 | Monolayer | Proprietary (Cruvinel et al., 2020) |

*Also differentiated using Protocol 4 (STEMdiff™ Cardiomyocyte Differentiation Kit from Stem Cell Technologies, according to the manufacturer’s instructions). MEF: mouse embryonic fibroblasts, hES: DMEM/F12, 20% KnockOut serum replacement, Glutamine, Non-essential aminoacids, β-mercaptoethanol and bFGF.
