# Supplementary material for: Action potential variability in human pluripotent stem cell-derived cardiomyocytes obtained from healthy donors
Source: Front Physiol. 2022 Dec 16;13:1077069. doi: 10.3389/fphys.2022.1077069 (PMC9800870; doi:10.3389/fphys.2022.1077069)
Supplement: Supplementary file 8 [file Table5.DOCX]

Suppl Table 5: Descriptive statistics for APA in mV per cell line

|  | Line 1 | Line 2 | Line 3 | Line 4 | Line 5 | Line 6 |
| --- | --- | --- | --- | --- | --- | --- |
| Min | 70.27 | 70.94 | 70.32 | 70.08 | 78.56 | 70.24 |
| 1^st^ Q | 78.05 | 83.33 | 77.00 | 83.67 | 93.26 | 81.89 |
| Median | 87.11 | 90.29 | 80.20 | 91.16 | 98.58 | 92.38 |
| 3^rd^ Q | 94.74 | 98.90 | 87.67 | 99.60 | 102.50 | 99.38 |
| Max | 107.9 | 119.0 | 111.7 | 125.0 | 109.9 | 109.4 |
| Mean | 86.85 | 91.09 | 82.66 | 92.26 | 98.02 | 90.84 |
| SD | 9.77 | 10.80 | 8.79 | 12.26 | 7.85 | 11.6 |

Min: minimum, 1^st^ Q: first quartile, 3^rd^ Q: third quartile, Max: maximum, SD: standard deviation.
