# Supplementary material for: Action potential variability in human pluripotent stem cell-derived cardiomyocytes obtained from healthy donors
Source: Front Physiol. 2022 Dec 16;13:1077069. doi: 10.3389/fphys.2022.1077069 (PMC9800870; doi:10.3389/fphys.2022.1077069)
Supplement: Supplementary file 9 [file Table3.DOCX]

Suppl Table 3: Number of cells analyzed according to cell type, cell line and differentiation protocol

| Cell type | Cell line | n | Differentiation protocol |
| --- | --- | --- | --- |
| ES (n = 138) | Line 1 | 138 | Protocol 1 |
| iPS (n = 642) | Line 2 | 196 | Protocol 2 (n = 180) |
|  |  |  | Protocol 4 (n = 16) |
|  | Line 3 | 43 | Protocol 2 |
|  | Line 4 | 347 | Protocol 2 |
|  | Line 5 | 27 | Protocol 2 |
|  | Line 6 | 29 | Protocol 3 |
| Total (ES + iPS) | | 780 |  |
