# Supplementary material for: Action potential variability in human pluripotent stem cell-derived cardiomyocytes obtained from healthy donors
Source: Front Physiol. 2022 Dec 16;13:1077069. doi: 10.3389/fphys.2022.1077069 (PMC9800870; doi:10.3389/fphys.2022.1077069)
Supplement: Supplementary file 10 [file Table4.DOCX]

Suppl Table 4: Descriptive statistics for MDP in mV per cell line

|  |  | Line 1 | Line 2 | Line 3 | Line 4 | Line 5 | Line 6 |
| --- | --- | --- | --- | --- | --- | --- | --- |
| Min |  | -87.87 | -86.31 | -93.35 | -94.00 | -67.75 | -71.39 |
| 1^st^ Q |  | -64.44 | -60.49 | -56.75 | -65.36 | -59.29 | -56.87 |
| Median |  | -57.66 | -54.99 | -51.82 | -60.27 | -55.92 | -54.29 |
| 3^rd^ Q |  | -50.51 | -49.71 | -47.42 | -53.83 | -50.79 | -50.94 |
| Max |  | -41.14 | -40.10 | -42.04 | -41.18 | -40.35 | -43.12 |
| Mean |  | -57.92 | -56.05 | -53.11 | -59.95 | -55.78 | -54.72 |
| SD |  | 9.38 | 9.12 | 9.42 | 9.14 | 6.57 | 6.58 |

Min: minimum, 1^st^ Q: first quartile, 3^rd^ Q: third quartile, Max: maximum, SD: standard deviation.
