# Supplementary material for: Action potential variability in human pluripotent stem cell-derived cardiomyocytes obtained from healthy donors
Source: Front Physiol. 2022 Dec 16;13:1077069. doi: 10.3389/fphys.2022.1077069 (PMC9800870; doi:10.3389/fphys.2022.1077069)
Supplement: Supplementary file 11 [file Table6.DOCX]

Suppl Table 6: Descriptive statistics for maximum dV/dt in mV/s per cell line

|  | Line 1 | Line 2 | Line 3 | Line 4 | Line 5 | Line 6 |
| --- | --- | --- | --- | --- | --- | --- |
| Min | 5.5x10^3^ | 6.3x10^3^ | 6.8x10^3^ | 4.9x10^3^ | 1.2x10^4^ | 4.3x10^3^ |
| 1^st^ Q | 1.0x10^4^ | 1.2x10^4^ | 1.0x10^4^ | 1.3x10^4^ | 2.2x10^4^ | 9.1x10^3^ |
| Median | 1.2x10^4^ | 1.6x10^4^ | 1.4x10^4^ | 2.1x10^4^ | 3.3x10^4^ | 1.1x10^4^ |
| 3^rd^ Q | 2.2x10^4^ | 3.0x10^4^ | 1.8x10^4^ | 3.5x10^4^ | 4.0x10^4^ | 1.4x10^4^ |
| Max | 8.2x10^4^ | 2.3x10^5^ | 3.6x10^4^ | 1.2x10^5^ | 6.0x10^4^ | 3.3x10^4^ |
| Mean | 1.8x10^4^ | 2.4x10^4^ | 1.5x10^4^ | 2.5x10^4^ | 3.2x10^4^ | 1.3x10^4^ |
| SD | 1.3x10^4^ | 2.4x10^4^ | 5.9x10^3^ | 1.6x10^4^ | 1.2x10^4^ | 6.7x10^3^ |

Min: minimum, 1^st^ Q: first quartile, 3^rd^ Q: third quartile, Max: maximum, SD: standard deviation.
